# The “Long Arm of Childhood” Influences Current Caregivers’ Health

**DOI:** 10.1093/geroni/igaf122.2983

**Published:** 2025-12-31

**Authors:** Payton Radcliffe, Julie Hicks-Patrick

**Affiliations:** West Virginia University, Morgantown, West Virginia, United States; West Virginia University, Morgantown, West Virginia, United States

## Abstract

Currently, 1 in 5 Americans are providing daily care to friends and family members. Informal caregiving exerts economic, physical, and emotional stress, often affecting health outcomes. We sought to understand whether supportive relationships in childhood influenced adult health and whether such buffers were more important for family caregivers than non-caregivers. We used the 2023 BRFSS (N = 9428; M_age=54.0, SD = 17.8; 52% females; 80.8% white nonHispanic), to test a moderated regression model examining the effects of unmet needs in childhood upon current physical health and whether these were altered as a function of current caregiver status (23.3% current caregivers). The moderated regression model assessing physical health was significant, F(3, 9424)=35.07***, R^2= .011. Direct effects of caregiver status (b = 18.6***) and lower unmet needs (b = 8.2***) were qualified by the interaction between the two (b=-2.6*). Post hoc analyses showed that caregivers whose childhood needs were never met reported more poor health days (M = 17.6) than non-caregivers whose needs were never met (M = 13.8), and more than all others whose basic childhood needs were met. Caregiving is difficult, but taking on that role may be more challenging for those whose childhood needs were not met. More attention is needed to lifespan contributors to caregiving.

